# Left ventricular hypertrophy in African children infected with HIV/AIDS: a case-control study

**DOI:** 10.11604/pamj.2023.45.110.37095

**Published:** 2023-06-29

**Authors:** Ijeoma Ogugua Arodiwe, Christopher Bismarck Eke, Ejikeme Benneth Arodiwe

**Affiliations:** 1 Department of Paediatrics, College of Medicine, University of Nigeria Enugu Campus, Enugu, Nigeria,; 2 Department of Medicine, College of Medicine, University of Nigeria Enugu Campus, Enugu, Nigeria

**Keywords:** Left ventricular, hypertrophy, children, retroviral infection, echocardiography

## Abstract

**Introduction:**

left ventricular hypertrophy (LVH) measured by echocardiography seen in human immunodeficiency virus/acquired immunodeficiency disease (HIV/AIDS) affects the morbidity and mortality. The hemodynamic and metabolic changes in (HIV/AIDS) affect the heart adversely causing hypertrophic remodeling with left ventricular hypertrophy. The aim of this study was to determine the prevalence and risk factors associated with LVH in African children with HIV/AIDS.

**Methods:**

an analytical case-control study was conducted using echocardiography to assess cardiac function. Descriptive statistics was used to determine percentages and univariate analysis to find association between dependent variable and independent variables. Independent variables that had an association in a univariate were included in the multivariate model to determine strength of association.

**Results:**

the mean age of the study population was 7.8 ± 2.07 years for controls and 8.3 ± 3.04 years for cases respectively. They were made up of 51.2% (n= 86) males and 48.8% (n = 82) females (M: F=1.05: 1). We studied eighty-four (n= 84) cases, and LVH was seen in 67.7% (n= 56) of the patients. Mean left ventricular mass index (g/m^2^) was significantly higher in the cases (90.37± 35.50) than controls (89.37 ± 14.25, p= 0.04.) Relative wall thickness (mm) was within normal in the control, 0.35 ± 0.06 and high in the cases, 0.67 ± 0.17, p= 0.01. Eccentric hypertrophy was the most common type seen in 36.9% (n= 31) of the patients. Multiple linear regression analysis, revealed that the presence of LVH was associated with 0.212 (95% CI: 0.001 - 0.014; p= 0.001) lower Body mass index (BMI) for age and 0.396 (95% CI; 0.002 - 0.066; p= 0.03) lower CD4+ cell count as predictors of LVH.

**Conclusion:**

the prevalence of LVH was high. Lower body mass index (BMI) and CD4+cells count predicted LVH. This supports the recommendation by the National Heart, Lung and Blood Institute (NHLBI) working group on research priorities for cardiovascular complications in HIV/AIDS, for baseline and periodic echocardiography in the management of children with HIV/AIDS.

## Introduction

Evidence of cardiac complications in HIV/AIDS may be clinically quiescent initially however, studies have reported that HIV/AIDS may exhibit a cardiac tropism and cardiac complications contribute significantly to morbidity and mortality in children [1,2]. The first report of cardiac involvement was by Autran *et al*. in Haiti in 1983 [3]. Although this tropism may involve all the cardiac tissues, the myocardium remains the most involved, showing evidence of dysfunctions. The most incongruous is left ventricular hypertrophy (LVH) in a disease mainly characterized by generalized wasting [1,2]. Ventricular dilatation in these children may be multifactorial and this causes compensatory hypertrophy resulting in significant increase in LV mass [4]. However, unlike in children without HIV/AIDS; the magnitude of hypertrophy is inadequate to counteract peak systolic wall stress to normal. Hence, the continued afterload excess leads to further use of preload reserve resulting in progressive ventricular dilatation. This continued elevation of peak systolic wall stress indicates that the degree of LV hypertrophy is inadequate for the progressively increasing LV dilatation resulting in cardiac failure [4]. The reported prevalence of left ventricular hypertrophy (LVH) in HIV infection from several studies in Europe and America varies from 2% to over 40% [5-7], with symptomatic heart failure developing in 6% of these patients most of who have end-stage disease, AIDS [6,7]. However, on LVH and its predictors on the African children, there are a few published controlled studies [8,9]. These noted increased left ventricular mass in 20.5% and 28% of the cases among the study population. They used LVM indexed to height (ht 2.7) and body surface area respectively. The need for indexing is important to remove the confounding effect of increasing body composition on increasing cardiac size in pediatrics population [8]. They noted both subclinical and overt cardiac abnormalities in HIV-infected African children and suggested routine echocardiography in these patients in standard care. Therefore, in a setting of limited resources, there is need that predictors of LVH be studied, as these may be useful in identifying the risk factors and provide baseline for monitoring patients for intervention. Some studies had noted that age, a low CD4+ cell counts, use of Highly Active Antiretroviral Therapy (HAART), malnutrition and wasting may predict LVH [10-12]. This present study aims at providing epidemiological data based on a comparative cross-sectional study design, will validate these factors of cardiac dysfunction in HIV/AIDS in our environment in a Nigerian urban city, using two-dimensional Doppler echocardiography.

## Methods

**Study design and setting:** this was an analytical case-control study carried out over a period of 12 months, August 2018 -July 2019, at the Pediatric Cardiology and Outpatient Pediatric HIV Clinic of the University of Nigeria Teaching Hospital (UNTH), Ituku/Ozalla, Enugu.

**Study population:** eighty-four (n=84) children with HIV/AIDS aged 18 months -14 years were sequentially recruited with (n = 84) seronegative healthy, age and sex-matched controls. The patients had positive HIV serology and a clinical diagnosis of HIV infection according to Centers for Disease Control and Prevention (CDC) criteria. They were classified into 2 groups: HIV-infected group with 70 patients and AIDS group with 14 patients. The controls were selected from the children´s out-patient (CHOP) department and immunization clinic of the same hospital. They were screened for HIV infection and were negative. The exclusion criteria for cases were children with pre-existing cardiac diseases, chronic diseases associated with demonstrable wasting or oedema. Informed written consents were obtained from parents/guardians of the children while assent was obtained from children 7 years and older respectively.

**Data collection:** the investigator administered the study pro forma to obtain demographic data and clinical history, including medication history and duration of HAART. All cases and controls were clinically examined by the investigator. Clinical assessment and echocardiography measurement obtained were entered in the pro forma and cardiac function calculated according to the recommendations of the American Society of Echocardiography was obtained [13]. Echocardiography technique and measurements: echocardiography study was done for the cases and controls by a single experienced certified pediatric cardiologist. Two-dimensional (2D), mode (M), and Doppler echocardiography examinations were performed with Hewlett-Packard SONO 2000 machine with a 5.5-12MHz multi transducer, wide phased array transducer. The investigators were blind to the HIV and clinical status of the study groups. The younger subject who was not cooperative in the presence of their caregiver or parents was pacified with toys or sedated with a mild short-acting sedative, chloral hydrate as appropriate. This was given half an hour before the procedure and aqua sonic gel used to ensure effective contact of the transducer with the chest wall. Older children were placed in a left lateral position and examined in the standard subcostal, apical, parasternal long and short axis and suprasternal views. Using the cross-sectional images as a guide, the M-mode tracing of the left ventricle was obtained and entered into the questionnaire to calculate measurements according to the recommendations of the American Society of Echocardiography [13].

**Laboratory analysis:** blood was drawn for hemoglobin (Hb) measurement and erythrocyte sedimentation rate (ESR), CD4+ cell count (performed by a slide technique with anti- alkaline phosphatase (APAAP) mouse monoclonal antibody (Dako Glostrup, Denmark) and HIV serology. Antibody level against HIV were measured by an enzyme linked immunosorbent assay and confirmed by western blotting.

**Definitions:** Left ventricular Mass (LVM) g = 1.05 [(LVEDd + LVPW + IVS)^3^- LVEDd^3^]–14.


$$\text{Left ventricular mass index }\left(\text{LVMI}\right){\text{g/m}}^{2.7}=\frac{\text{LVM}\left(g\right)}{\text{Height }{\left(\text{m}\right)}^{2.7}}$$

Adjustment was made for the confounding variable of changing body size with age, by indexing the LVM to height in meters raised to power of 2.7. Relative wall thickness (RWT) = 2 (LVPW)/LVEDd. LVEDd: interventricular end-diastolic dimension; LVPW: left Ventricular posterior wall; IVS: interventricular septum. Left ventricular hypertrophy means a cut-off value of LVMI of 103g/m^2^ for males and 84g/m^2^ for females. Further characterization of LVH into concentric and eccentric was done using increased RWT defined as RWT ≥ 0.42. Concentric LVH is present when both RWT and LVM are elevated while eccentric LVH is when LVM is elevated with a normal RWT [14].

**Study size:** sample size estimation. The sample size was calculated using Fisher’s formula.


$$n=\frac{Z^{2}\left(pq\right)}{d^{2}}$$

Where n = minimum sample size, z = 95% confidence interval i.e. (1.96), P = median prevalence of cardiovascular involvement in HIV (12.5%), d = level of precision (0.075), q= 1-p, n= 75 subjects. Addition of an attrition rate of 10% brought the sample size to 83.


$$n=\frac{1.96^{2}\times 0.125\left(1-0.125\right)}{0.075^{2}}$$

**Statistical analysis:** statistical package for social sciences (SPSS) version 20.0 was used to enter and analyze the data. Descriptive statistics for continuous variables were expressed as mean (SD) and the means were compared by using analysis of variance or two sample t-test. Categorical qualitative variables were presented as percentage or frequencies and compared between the HIV - infected, AIDS and controls groups by using the nonparametric test of association between the groups using one-way analysis of variance (ANOVA) or chi-square test as appropriate. A p-value < 0.05 was considered statistically significant. Pearson´s and Spearman Rho correlation were used to assess the relations between LV hypertrophy as dependent variable and independent variables affecting it. Significant variables were further analyzed using the stepwise linear regression analysis to isolate possible determinants of LVH.

**Ethical considerations of consent:** ethical approval was obtained from the Ethical Committee of the University of Nigeria Teaching Hospital, Ituku- Ozalla, Enugu, with approval number NHREC/05/01/2018B. To participate in the study, informed consent and assent were obtained from parents/guardians of the children and older children respectively. They were duly educated on the nature of the procedure, their voluntary participation or otherwise and the possible benefits of the study.

## Results

**General characteristics of the study population:** eighty-four cases and eighty-four age and sex-matched controls were consecutively recruited for the study. For the cases, 69 were HIV infected and 15 had category C disease (AIDS). The mean age of the cases (children with HIV/AIDS) was 8.3 ± 3.04 (range 2-14 years) with a male to female (M: F) ratio of (1.05: 1), while the mean age of the controls was 7.9 ± 2.07 (range 2 -13 years) with a male: female ratio of (1.19: 1). There is no significant difference between them in terms of age and sex (p = 0.34; 0.14) respectively (Table 1). There was a significant difference among the cases and controls in the following parameters: the cases were lighter with a mean BMI of 15.3 ± 0.5kg/m^2^, had increased HR of 121 ± 20 breaths/min and ESR of 67±12.4mm/1^st^ hr, and they also have lower CD4+ cell count of 504.6 ± 300.3 cell/mm^3^. There was a significant difference in the prevalence of LVH between the cases with 66.7% (n= 56) and the controls having only 4.8% (n= 4). The eccentric type 36.9% (n= 31) is the most common type (χ^2^ 14.3, p = 0.02). Most of the cases 85.7% (n=72) were on HAART with zidovudine-based combination (Table 2).

**Table 1:** characteristics of the study participants

Characteristics	Controls n = 84	Cases n = 84	F/χ2	p - value
Mean age (years)	7.9 ± 2.07	8.3 ± 3.04	0.12	0.34
Sex ratio (m: f)	1.19: 1	1.05:1	6.80	0.14
Mean BMI for age (kg/m^2^)	21.4 ±0. 3	15.3 ± 0.5	94.11	0.02
Mean RR/min	24±5	32±6	13.12	0.01
Mean HR/min	90±13	121±20	25.0	0.001
ESR (mm/1^st^ hr)	6.3 ± 2.4	67±12.4	48.40	0.01
Mean CD4+ (cells/mm^3^)	1786.6 ±1582.6	504.6 ± 300.3	8.93	0.001
≤ 1499, n (%)	10 (11.9)	79 (94.0)	5.60	0.01
≥ 1500, n (%)	74 (88.1)	5 (5.9)	4.54	0.05
Treatment with HAART {n (%)}				
Yes	0 (0)	72 (85.7)	-	-
No	0 (0)	12 (14.2)	-	-
RWT (mm)	0.35 ± 0.06	0.67 ± 0.17	1.21	0.01
LVMI (g/m^2^)	89.37 ± 14.25	90.37± 35.50	1.59	0.04
Ejection fraction (%)	53.33± 15.73	45.33± 12.73	0.70	0.02
Prevalence of hypertension (%)	2.0 (2.5)	45 (53.5)	7.76	0.001
Prevalence of LVH (%)	4. 0 (4.8)	56 (66.7)		
Concentric	4.0 (4.8)	25 (29.7)		
Eccentric	0	31 (36.9)	14.3	0.02
No LVH	77. 0 (95.2)	28 (33.3)		

BMI: body mass inde; RR: respiratory rate, HR: heart rate; LVMI: left ventricular mass index; IVS: interventricular septum; LVEDd: left ventricular end diastolic dimension; RWT: relative wall thickness; LVH: left ventricular hypertrophy

**Table 2:** correlation of independent variables with left ventricular hypertrophy in HIV infection and AIDS groups

	HIV infection			AIDS		
Independent variable	Correlation coefficient (r)	95% CI	P-value	Correlation coefficient (r)	95% CI	p-value
Age (years)	0.32	0.12, 0.53	0.23	0.22	0.09, 0.25	0.11
BMI for – age	0.19	0.11, 0.32	0.03*	0.20	0.09,0.25	0.01*
Duration of treatment (years)	- 0.49	-0.33, 0.55	0.01*	-0.45	-0.30, 0.51	0.07
SBP (mmHg)	-0.29	- 0.11, 0.35	0.12	-0.30	-0.10, 0.46	0.45
DBP (mmHg)	-0.38	- 0.20, 0.65	0. 04*	-0.35	-0.20, 0.57	0.53
Hemoglobin conc (g/dl)	-0.20	- 0.08, 0.33	0.30	-0.25	-0.10, 0.43	0.62
WBC (total)	0.01	0.01, 0.09	0.95	-0.05	-0.01, 0.09	0.12
ESR	-0.33	-0.11, 0.45	0.08	-0.35	0.02, 0.42	0.24
CD4+cell count	0.08	0.04, 0.19	0.01*	0.09	0.03,0.12	0.02*
Pulse rate	-0.13	0.10, 0.19	0.04*	-0.15	- 0.11, 0.03	0.03*

* significant

**Correlation analysis:** in the HIV infection group, significant correlation was noted with LVH and the following variable; BMI for age r= 0.19, CI = (0.11, 0.32), duration of treatment r = -0.49, CI = (-0.33, 0.55), diastolic blood pressure r = -0.38, CI = (-0.20, 0.65), CD4+ cell count r = 0.08, CI = (- 0.01, 0.12), and pulse rate r = -0.13, CI = (0.10, 0.19) (Table 2). In the AIDS group, there was significant correlation with LVH and lower BMI adjusted for age r = 0.20, CI= (0.09, 0.25) and lower CD4 cell count r = 0.09, CI= (0.03, 0.12) and increased pulse rate r = -0.015, CI= (- 0.01, 0.03) (Table 2).

**Multivariate regression analysis:** multiple linear regression analysis, revealed that the presence of LVH was associated positively with lower BMI for age with beta coefficient of 0.212 (95% CI: 0.001 - 0.014; p = 0.001) and lower CD4+ cell count with beta coefficient of 0.396 (95% CI; 0.002 - 0.066; p= 0.03). These best predicts the presence of LVH (Table 3).

**Table 3:** stepwise multiple linear regressions of factors that correlated with left ventricular hypertrophy in the study population

Model	Standardized coefficients (r)		T		p-value	95% CI for B	
	Std. error	Beta					
Body mass index for age	0.051	0.212		1.170	0.01*	0.001-	0.014
CD4+ cell counts	0.016	0.396		2.186	0.03*	0.002-	0.066

a: Dependent variable; *: significant

## Discussion

We present echocardiography result of children with HIV/AIDS at University of Nigeria Teaching Hospital, Enugu in South-East, Nigeria. This index study showed that HIV/AIDS is associated with increased LV mass index and LVH when compared to controls, with the prevalence of LVH been at 66.7%. This had been reported nationally [8,9] and internationally [15-18] but with different prevalence. Ours was higher than 21% and 29% that was noted in Nigerian [9] and Indian [15] children respectively. However, it was lower than 67% seen in Zimbabwean children [16]. These differences may be due to diagnostic criteria used or true geographic variations in the epidemiology. For example on the diagnostic criteria; while the index study used LVM indexed to height raised to the power of 2.7, the Nigerian study used LVM indexed to body surface area [9], the Zimbabwean work used LVM alone without indexing [16]. The need for indexing with height is important to remove the confounding effect of increasing body composition on increasing cardiac size seen in pediatrics population [8]. These differences may be true variations in epidemiology of cardiac dysfunction in HIV/AIDS. This has led to the consensus that the frequency of cardiac dysfunctions depends on the epidemiology of the population being studied. Hence, the advocacy on research priorities for cardiovascular complications in HIV/AIDS among population by the National Heart, Lung and Blood Institute working group [19]. As this index study is the only known study from this area; its overall result is native and significant and supports the above research priorities in these children. Left ventricular hypertrophy in this index study were noted to occur in the younger age below seven years. Other studies have documented similar finding [8,20]. This age group may be at higher risk for HIV-associated cardiac abnormalities for these reasons. The vertical transmission of HIV infection; which occurs in utero or at delivery may have caused cardiac developmental abnormality or given ample time for the virus to cause changes on the myocytes [20].

This mode of transmission of HIV is still the most common in developing countries due to low coverage of prevention of mother to child transmission (PMTCT) services [8]. Also, the immaturity of cardiac structures associated with younger age and the use of HAART are possible reasons for this observation [8]. Left ventricular hypertrophy is a useful predictor of mortality risk in children with HIV-infection/AIDS [20]. This risk is higher with concentric LVH type. However, this was reported in an adult study [21]. This index study noted eccentric type as the most common type in 36.9%. The mortality outcome was not documented in this index study, there may be some differences in the children population. However, the risk of death is higher in children in most developing countries because of late presentation to health facilities due to poverty and lack of supportive health system structure, like health insurance [22]. This may negatively confound the mortality risk in these children. The mean BMI for age was significantly lower in HIV/AIDS groups, as shown in Table 1. This is expected as the loss of lean body mass especially muscle protein has been documented in HIV-infection [23]. However, LVH in some of the patients with HIV infection, as seen in this index study and others [8,9,16,20], is paradoxical and of research interest. Possible explanations are the effect of proto-oncogene activation seen in retroviruses infection, counteracting the usual effects of malnutrition on cardiac muscle leading to hypertrophic changes [24,25]. Also in these children there is additional progressive ventricular dilatation [26,27]. Ventricular dilatation in HIV infection results from possibly multifactorial causes as chronic anemia, volume overload and cardiotoxicity from HAART. This may also evoke the normal sequence of compensatory hypertrophy [27]. The finding in this index study suggests that the stimulus for LVH is the lower BMI for age, which may have triggered the paradoxical effect of hypertrophy. The lower BMI for age best predicts LVH followed by CD4+ cell count, as seen in Table 3. Although cardiac abnormalities tend to be prevalent with declining immune status; CD4+ cell count has been shown by some studies to be a poor indicator of cardiovascular dysfunction [28-30]. This conflicting finding may be due to the timing of the study. In the pre HAART era it was so [29] but now it is showing a different pattern [28,30].

Also, the cut-off point of CD4+ counts used in these studies are different. This may lead to variation in result. Drug treatment with HAART is reported to have varying effect, ranging from cardio protection to cardiotoxicity [29,31]. Some drugs used in HAART such as zidovudine (AZT) and abacavir (ABC) have been noted to have cardiotoxicity effect. This was also observed in this index study, as cases on this regimen showed higher prevalence of LVH as shown in Table 1. The links between HAART and LVH have been reported [29,30]. The authors reported LV dysfunction in HIV-infected patients on HAART, especially AZT [29,30]. Other workers [8,31], had reported that AZT has no effect on cardiac muscles. However, some suggests that HAART is generally cardio-protective albeit, this protective effect is more pronounced in the early childhood and wean over time [31,32]. Therefore, response of any index patient may depend on the effect of the confounding factors. Again, HIV/AIDS is an emerging disease and so is drug pharmacodynamics. However, a prospective study of subjects at commencement of HAART and longitudinal follow up would be a better study design for this evaluation.

The strength of the study includes use of analytical case control study design with matching of cases and controls. The echocardiography was done by the same cardiologist, minimizing inter-observer bias. Also, the selection bias inherent in research from referral centers was considered in the sample composition, as the authors studied all eligible children. The weakness lies in the sampling method being a non-probability convenience sample. This limits the external validity of the results.

## Conclusion

The present study showed that LVH prevalence is high at 66.7%. Lower BMI and CD4+ cells count predicted this high prevalence and it was common in younger age below seven years. These parameters can be used as criteria for selection of these patients for periodic evaluation with echocardiography in resource challenged countries. This will help early detection of cardiac dysfunction and prevent the risk of mortality. Again this study concludes by supporting recommendation for baseline and periodic echocardiography in the management of children with HIV/AIDS. Finally, there is also need for local ratification of reference values for pediatric echocardiographic variables using World Health Organization (WHO) standards z-scores.

### What is known about this topic

- *HIV/AIDS affects the heart and best practice in developed world support baseline and routine echocardiography in patients’ management for early detection;*
- *This HIV-linked cardiac dysfunction such as LV systolic dysfunction and LVH are initially clinically quiescent but later are cardiovascular risk in these children;*
- *Both concentric and eccentric types of LVH have been noted in children with HIV/AIDS*.

### What this study adds

- *There is a dearth of analytical case control studies on HIV/AIDS and cardiac function in sub-Saharan African; this study showing cardiac dysfunctions in African children with HIV/AIDS fills the gap;*
- *This index study is the only work done in the South East of Nigeria in Africa, using LVM indexed to height raised to the power of 2.7, (height 2.7) to calculate LVH; this gives accurate assessment;*
- *The use of analytical case control study design with inferential statistics gives a strong epidemiological data and showed a high prevalence of LVH as reported in developed countries; finally, this strengthens the recommendation for baseline and periodic echocardiography in the management of children with HIV/AIDS*.
